# Mesenteric panniculitis presenting with acute non-occlusive colonic ischemia

**DOI:** 10.1186/1755-7682-4-22

**Published:** 2011-06-22

**Authors:** Florian Amor, Mohsen Farsad, Romano Polato, Patrizia Pernter, Josef Widmann, Guido Mazzoleni, Luzian Osele, Christian J Wiedermann

**Affiliations:** 1 Division of Internal Medicine, Central Hospital of Bolzano, Lorenz Böhler Street 5, Bolzano 39100, Italy; 2 Division of Nuclear Medicine, Central Hospital of Bolzano, Lorenz Böhler Street 5, Bolzano 39100, Italy; 3 Division of General Surgery, Central Hospital of Bolzano, Lorenz Böhler Street 5, Bolzano 39100, Italy; 4 Division of Radiology, Central Hospital of Bolzano, Lorenz Böhler Street 5, Bolzano 39100, Italy; 5 Division of Pathological Anatomy and Histology, Central Hospital of Bolzano, Lorenz Böhler Street 5, Bolzano 39100, Italy

**Keywords:** *panniculitis*, *peritoneal*, *colitis*, *ischemic*, *positron emission tomography*

## Abstract

**Background:**

The role of positron emission tomography (PET) of the mesentery as a diagnostic modality in cases of mesenteric panniculitis is unclear.

**Case presentation:**

A 67-year-old woman presented with rectal bleeding due to nonocclusive colonic ischemia. Abdominal CT showed features of mesenteric panniculitis. PET-CT demonstrated no abnormal fluorine-18 fluordeoxyglucose uptake in the affected mesentery or any surrounding lymph nodes. Laparoscopic biopsies from a thickened segment of mesenteric fat excluded neoplastic infiltration.

**Conclusions:**

In cases of unexplained ischemic colitis, panniculitis should be considered a possible diagnosis. PET-CT may be negative for fluorine-18 fluordeoxyglucose uptake in this condition. As of known false-negative PET-CT results in mesenteric panniculitis, PET-CT has a limited role in the diagnostic work-up.

## Background

Mesenteric panniculitis (MP) is an acute benign fibrosing and inflammatory condition that involves the adipose tissue of the mesentery. In most patients, the condition consists of a mixture of mesenteric fat necrosis (lipodystrophy), chronic non-specific inflammation (panniculitis) and fibrosis (sclerosing mesenteritis) of unknown etiology. Several mechanisms underlying this condition have been suggested based upon anecdotal reports, small case series, or extrapolation from animal models of inflammation. These include previous abdominal surgery or trauma, autoimmunity, paraneoplastic syndrome, ischemic injury, and infection. Most patients are in the fifth to the seventh decades of life, with the median age of patients being 65 years. Clinical manifestations are nonspecific and atypical. Patients may present with abdominal pain, intestinal obstruction, fever, chylous ascites, a mass, constipation or diarrhoea; bleeding has also been reported [1]. Abdominal CT is the most sensitive imaging modality for detecting MP. However, its specificity is limited since a broad differential diagnosis exists for mass lesions of the mesentery, in particular for mesenteric tumoural involvement [1,2].

According to one report, the combination of positron emission tomography (PET) scanning and CT is helpful in differentiating MP from MP co-existing with tumor since in patients only with MP, CT was positive for MP, but PET scanning was negative [3]. The authors therefore concluded that a negative PET excludes tumoral involvement with high accuracy. However, it has also been reported that fluorine-18 fluorodeoxyglucose (FDG) PET was positive in sclerosing mesenteritis with no tumoral involvement [4], so that the specificity of PET in diagnosing mesenteric tumors is unclear.

The objective of this case report is to present an additional case of MP with reversible ischemic colonopathy that was negative in FDG PET with multidetector CT correlation.

## Case presentation

A 67-year-old female Caucasian patient was admitted to our hospital with a one-day history of rapid onset of severe left lower abdominal pain and tenderness, which was associated with nausea and vomiting with frequent passage of bloody, loose stools. Ten days earlier, because of gingivitis, amoxicillin-clavulanate had been prescribed for one week that was associated with episodic abdominal discomfort that lasted a few minutes. Episodic abdominal pain was already present during the preceding 3 months but was not associated with other symptoms such as bloating, diarrhoea, constipation, nausea, vomiting, early satiety, weakness, fever/chills or anorexia; the discomfort could not be ignored but did not influence daily activities (Mesenteric Panniculitis Subjective Assessment Score MPSAS [5] of 16) suggestive of moderately symptomatic disease.

Her past medical history included hypertension and dyslipidemia for 10 years and impaired glucose tolerance diagnosed only recently. Urinary incontinence due to childbirth-related pelvic floor defect and uterine descent was surgically corrected 3 years ago. Her medication history included daily clonidine 0.3 mg divided in two doses, lercadipine 20 mg, olmesartan 20 mg, hydrochlorothiazide 20 mg, rosuvastatin 10 mg, and acetylsalicylic acid 75 mg. She had no known allergies, no significant family history, and a review of her systems was unremarkable.

On examination, the patient was in sinus rhythm, was moderately dehydrated, had lower abdominal tenderness, and had no organomegaly or palpable masses. Body mass index was 33.9 kg/m^2^. Digital rectal examination results were positive for blood. Laboratory tests showed moderate leukocytosis (white blood cell count, 15, 700/μL; with an elevated neutrophil count of 13, 500/μL), an elevated C-reactive protein level (67 mg/L, normal range < 5 mg/L), and an erythrocyte sedimentation rate of 39 mm/h. Arterial blood gas showed no lactic acidosis. The patient was admitted for investigation.

Chest and abdominal plain X-rays, as well as abdominal ultrasonography were normal. Stool cultures for *Salmonella, Shigella, Campylobacter *and *Escherichia coli *O157:H7 and Clostridium difficile toxin A and B were negative. Complete ileo-colonoscopy performed within 48 hours of presentation showed petechial hemorrhages interspersed with areas of pale, edematous mucosa in the distal sigma and at the splenic flexure with rectal sparing. No strictures, diverticula, polyps, neoplastic tissue or ulcera were detected. Multiple biopsy specimens were taken and histology was consistent with minimal changes related to a ischemic colitis demonstrating diffuse hyperaemic dilated blood vessels free of thombi associated with minimal eosinophilic edema and a light chronic and acute mucosal inflammation. Antinuclear, antineutrophilic cytoplasmic, antiphospholipid and anti-double-stranded DNA antibodies were negative. Antithrombin and protein C plasma levels were normal; factor V Leiden and prothrombin gene mutations were negative. Therapeutic administration of acetylsalicylic acid was interrupted and enoxaparin 40 mg subcutaneously administered instead; metronidazole 500 mg every eight hours was given orally for suspected infectious colitis. Rectal bleeding stopped and acute abdominal pain significantly improved with no need of additional analgesic therapy.

**Figure 1:**
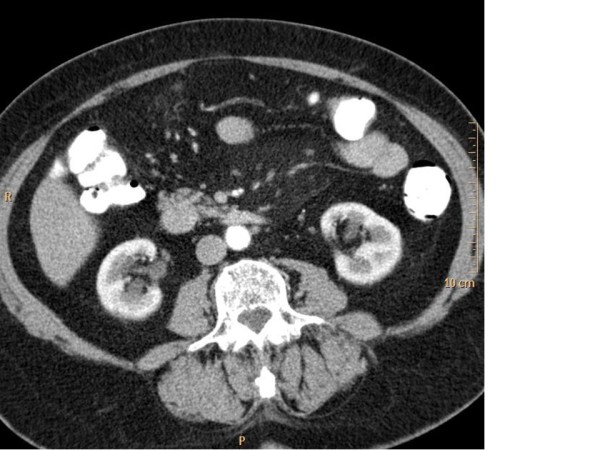
**CT findings of mesenteric panniculitis**. Enhanced abdominal CT at the mid-abdomen shows a hypertrophied fatty tissue in the mesenteric root with slightly increased attenuation delimited by a linear hyperdense rim (pseudo capsule). Some small nodules along the mesenteric vessels corresponding to lymph nodes but no mesenteric nodules of solid non fatty tissue.

**Figure 2:**
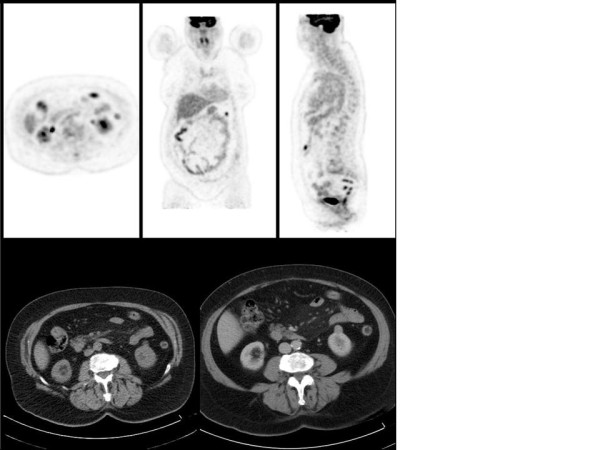
**No abnormal areas of uptake on PET scan in the mesenterium**.

On day 5 after admission, contrast-enhanced CT was performed which showed unremarkable results for small intestine, colon, liver, spleen, pancreas adrenal glands and pelvic organs. It revealed an area of mesenteric panniculitis at the root of the small bowel mesentery (Figure 1). Vascular contrast CT findings of mesenteric arteries were normal, thus excluding luminal thrombosis, vasculitis, abdominal aortic aneurysm and dissection or strangulation of the mesentery as typical causes for both, occlusive and non-occlusive types of ischemic colitis. Slightly increased vascular markings in projection to the proximal transversal mesocolon, compatible with venous stasis, were observed. A repeat CT scan performed 2 months later showed no appreciable change in the size of MP. In PET/CT imaging, no FDG uptake was seen within typical features of MP (Figure 2).

**Figure 3:**
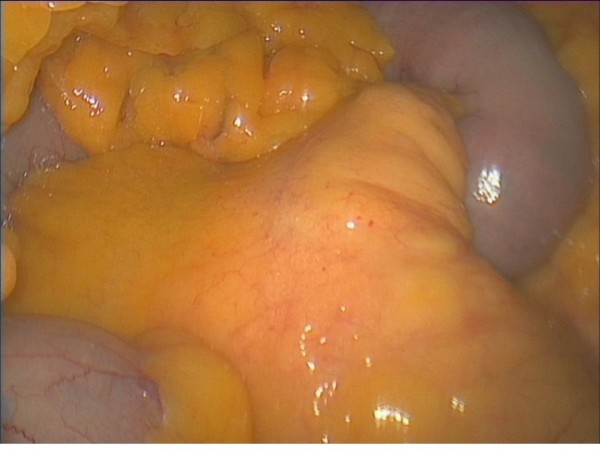
**Amorphous thickening of the mesenteric fat, which did not invade into the adjacent organs, visualized during laparoscopic biopsy**.

Laparoscopy was performed 1 month later demonstrating a slight whitish discoloration of the yellow upper mesenteric fat in proximity to the ligament of Treitz (Figure 3). Biopsies from the mesenteric surface of this area were taken; histology, however, was basically unremarkable with evidence of some submesothelial inflammation only.

After initial presentation, preexisting symptoms persisted for another two months and then gradually disappeared (MPSAS of 10). She had no abdominal pain any more, no change in bowel habits or hematochezia and no weight loss.

An exophytic renal mass of the left kidney of 15 × 17 × 18 mm size had already been noted in the first CT scan, which was confirmed in PET-CT imaging as not taking up FDG. A repeat CT scan after 14 months showed that the area of MP was slightly reduced. A small increment in size of this renal mass (17 × 17 × 20 mm) was noted and therefore the tumor was laparoscopically enucleated. Pathologic examination of the specimen revealed an oncocitoma with no coexistence of renal carcinoma and negative surgical margins.

## Conclusions

MP is most often an incidental finding during an investigation for other reasons. The most common physical finding is a poorly defined mass or masses in the right upper quadrant. Patients usually present with vague symptoms of abdominal fullness and pain in the upper or central abdomen [1]. There may also be nausea, altered bowel habit and lethargy. Rectal bleeding, as in the patient presented here, is an unusual presentation for MP [6]. Even though in our patient hematochezia could have been caused by antibiotic-associated colitis because of a preceding course of therapy with a broad-spectrum penicillin, histological examination of colonic biopsies and microbiologic testing excluded the differential diagnosis of Clostridium difficile-associated colitis.

Laboratory investigations are usually grossly normal. Studies found that patients had a raised erythrocyte sedimentation rate or other acute phase reactants [1]. In our case, elevated laboratory signs of inflammation were found only during the acute phase of rectal bleeding and likely due to ischemic colitis as all inflammation laboratory parameters normalized after the acute phase and remained normal throughout the observation period during which MP, though asymptomatic, was present radiologically.

Imaging is perhaps the most useful non-invasive investigation for MP with at least one published study suggesting that CT changes are so classical that the diagnosis can be made from this imaging modality alone [1]. In our patient, ultrasound examination and plain abdominal X-ray were not diagnostic. The CT changes seen in MP depend in large part upon the stage of the disease and whether the disease process is inflammatory mesenteric panniculitis or fibrotic mesenteric panniculitis [7]. In general, CT changes consistent with MP include encapsulated, heterogeneous masses localised to the root of the mesentery or adjacent intestinal loops [7]. Most patients have a left-sided orientation of disease with scattered, well-defined soft tissue nodules of < 5 mm [7]. Many have a 'pseudotumoral stripe' of tissue surrounding the mass lesion which may be seen in conjunction with mesenteric vessels which are surrounded or displaced by fat but not invaded [8]. Even though typical CT features of MP were seen in our case, mesenteric vessels were not clearly surrounded by nodule tissue despite rectal bleeding on initial presentation.

Given the protean clinical manifestations and nonspecific laboratory and radiologic findings, the diagnosis of MP is rarely made preoperatively. CT findings such as in our case are nonspecific and are generally not recommended for ruling out the coexistence of a neoplastic process such as lymphoma. A surgically obtained biopsy is required to confirm the diagnosis of MP [1,9]. PET-CT imaging may be used to differentiate between benign and neoplastic processes of the mesentery. Zissin et al. [3] examined 19 patients with known malignancy and incidental findings of mesenteric panniculitis on CT scan with PET-CT evaluation. The absence of FDG uptake within the areas of panniculitis in 11 of the 19 patients was found to be indicative of a non-neoplastic process after clinical evaluation and follow-up. No false-negative results were reported. A PET-CT scan ordered in the present case 2 months after admission because of initial rectal bleeding to rule out malignancy of both mesenteric and exophytic renal masses demonstrated no abnormal FDG uptake in the affected mesentery or any surrounding lymph node as also no renal uptake. These results are in line with observations reported [3]. However, a recently published case demonstrates that, in patients presenting with a mesenteric mass without a history of malignancy, PET-CT alone may miss the diagnosis of non-FDG avid malignancy, as biopsy of the mesenteric mass by laparoscopy identified low-grad lymphoma [9]. Independent of the results of PET-CT scanning, it is advisable to confirm the diagnosis of MP and to rule out a neoplastic process histologically. Laparoscopic biopsies from a thickened segment of mesenteric fat successfully ruled out malignancy in the present case.

Treatment of MP is empirical and based on a few selected drugs. In general, therapy has been reserved for symptomatic cases whereas incidental masses may be observed and left untreated. A wide variety of drugs including steroids, thalidomide, cyclophosphamide, progesterone, colchicine, azathioprine, tamoxifen, antibiotics and emetine, or radiotherapy are used with different degrees of therapeutic success with surgery reserved for cases in which medical therapy fails or in the presence of life-threatening complications such as bowel obstruction or perforation [10]. Our patient was treated with metronidazole for suspected infectious colitis; no anti-inflammatory drugs were given since the patient was only slightly symptomatic after rectal bleeding had stopped. The persisting and subsequently asymptomatic mesenteric mass was left untreated.

In conclusion, a PET-CT scan was negative in a patient presenting de novo with mesenteric abnormalities subsequently characterised as MP by laparoscopy and histopathology. When ruling out malignancy in CT-detected mesenteric masses, unremarkable results in PET-CT do not exclude neoplastic origin of the alterations in the mesentery, thus, confirming the limited diagnostic role of PET in this condition. In addition, MP should be considered a possible diagnosis in cases of unexplained ischemic colitis.

## Consent

Informed consent was obtained from the patient for publication of the case report.

## List of abbreviations

FDG: fluorine-18 fluorodeoxyglucose; MP: mesenteric panniculitis; MPSAS: Mesenteric Panniculitis Subjective Assessment Score; PET: positron emission tomography.

## Conflict of interest disclosure

The authors declare that they have no competing interests.

## Authors' contributions

FA and CJW were involved in writing the paper with data collection and literature search. MF, RP, PP and JW, registrars, were involved in treating the patient and data collection. GM and LO, consultants, were involved in treating the patient.
